# Early Origins of Autism Comorbidity: Neuropsychiatric Traits Correlated in Childhood Are Independent in Infancy

**DOI:** 10.1007/s10802-018-0410-1

**Published:** 2018-03-16

**Authors:** Zoë W. Hawks, Natasha Marrus, Anne L. Glowinski, John N. Constantino

**Affiliations:** 1 Department of Psychological & Brain Sciences, Washington University, One Brookings Drive, St. Louis, MO 63130 USA; 2 Department of Psychiatry, Washington University School of Medicine, 660 S. Euclid Avenue, St. Louis, MO 63110 USA; 3 Department of Pediatrics, Washington University School of Medicine, 1 Children’s Place, St. Louis, MO 63110 USA

**Keywords:** Autism, Psychopathology, Twins, Trait overlap, Development

## Abstract

Previous research has suggested that behavioral comorbidity is the rule rather than the exception in autism. The present study aimed to trace the respective origins of autistic and general psychopathologic traits—and their association—to infancy. Measurements of autistic traits and early liability for general psychopathology were assessed in 314 twins at 18 months, ascertained from the general population using birth records. 222 twins were re-evaluated at 36 months. Standardized ratings of variation in social communication at 18 months were highly heritable and strongly predicted autistic trait scores at 36 months. These early indices of autistic liability were independent from contemporaneous ratings of behavior problems on the *Brief Infant-Toddler Social and Emotional Assessment* (which were substantially environmentally-influenced), and did not meaningfully predict internalizing or externalizing scores on the *Achenbach Scales of Empirically Based Assessment* at 36 months. In this general population infant twin study, variation in social communication was independent from variation in other domains of general psychopathology, and exhibited a distinct genetic structure. The commonly-observed comorbidity of specific psychiatric syndromes with autism may arise from subsequent interactions between autistic liability and independent susceptibilities to other psychopathologic traits, suggesting opportunities for preventive amelioration of outcomes of these interactions over the course of development.

**Electronic supplementary material:**

The online version of this article (10.1007/s10802-018-0410-1) contains supplementary material, which is available to authorized users.

Ever since it was established that the characterizing features of the autistic syndrome were continuously distributed in the human population (Constantino and Todd 2003a; Ronald and Hoekstra 2011), and that the genetic factors which influence population variation in autistic traits overlapped with that of autism itself (Ronald et al. 2006; Constantino 2014), the exploration of how these traits influence general human social and behavioral development—specifically, the development of internalizing and externalizing behaviors, which are present in or characterize a wide array of psychiatric syndromes—has risen to high priority in behavioral neuroscience. The link between autistic traits and psychopathologic abnormalities of human behavior is reinforced by clinical studies indicating that a) within individuals, autism spectrum disorders frequently co-occur with behavioral disability (Dworzynski et al. 2009); b) autistic symptom severity is correlated with the severity of non-autistic behavioral impairments (Constantino and Frazier 2013); and c) within families, some of the genetic influences on the causation of ASD may be non-specific and therefore overlap with genetic influences on other psychiatric conditions of childhood (Constantino 2017; Mous et al. 2017).

The latter became apparent in genetic epidemiologic studies of school-aged children in which significant phenotypic associations were observed between traits that characterized autism and those that characterized attention-deficit/hyperactivity disorder (ADHD; Constantino et al. 2003b; Reiersen et al. 2007; Ronald et al. 2008; Ronald et al. 2010) and internalizing disorders (Hallett et al. 2012; Hallett et al. 2010; Duvekot et al. 2017). Additionally, within individual subjects, clinical studies have observed high comorbidity between autism spectrum disorder (ASD) and general psychopathology (Lundström et al. 2015; Simonoff et al. 2008; Hallett et al. 2013), with 70–96% of ASD diagnoses complicated by at least one co-occurring disorder (Simonoff et al. 2008; Lundström et al. 2015).

Studies of rare, highly-penetrant mutations associated with neuropsychiatric syndromes commonly reveal the phenomenon of pleiotropy, in which a given disease-causing variant can result in different neurobehavioral syndromes depending on the genetic background on which it is superimposed (Geschwind 2011; Moreno-De-Luca et al. 2013). Extending these findings, recent research suggests that common variants can also exert pleiotropic influences, constituting nonspecific etiologic factors that increase risk for symptoms of disparate psychopathologies (Lahey et al. 2017). Thus, phenotypic and genetic overlap between two inherited neuropsychiatric conditions is theoretically attributable to a) genetic influence shared by the conditions, and/or b) interactions over the course of development that result in one condition exacerbating the severity or manifestations of the other when (and only when) symptoms of both are present (Angold et al. 1999). Critically, studies of trait overlap in the general population can inform our understanding of clinical comorbidity if—as is the case for autistic traits—the measured subclinical traits are continuously distributed and causally associated with their more severe manifestations as clinical syndromes (Robinson et al. 2011).

An approach to resolving questions about the mechanisms contributing to overlapping phenotypes is to trace the respective domains to their developmental origins in infancy within a genetically-informative research design. To our knowledge, only two studies have investigated genetic influences on trait overlap between autism and general psychopathology in twins or families prior to school age, and none have investigated genetic influences on trait overlap prior to 2 years of age. Ronald et al. (2010) evaluated associations between ratings on the Child Behavior Checklist (CBCL) Pervasive Developmental Problems Scale (an index of quasi-autistic symptomatology; Achenbach and Rescorla 2000) and ADHD Scale (CBCL ADHD) in a community sample of 2-year-old twins, observing modest phenotypic correlations (*r* = 0.23–0.26) and shared genetic influences (genetic correlation = 0.27). The utility of the CBCL for ascertaining the characterizing traits and features of autism is somewhat limited, however, because it only captures a fraction of the variance captured by more specific assessment of autistic traits (see Bölte et al. 2008). Micalizzi et al. (2016) used a cross-lagged twin design to evaluate the time course of associations between autistic traits (here again, the CBCL Pervasive Developmental Problems scale was used) and affective problems (CBCL Affective Problems scale) during the third year of life. They observed substantial correlations between autistic traits and affective problems, which were attributable to shared and nonshared environmental influences at age 2 and genetic influences at age 3. In contrast with findings from older children (Hallett et al. 2010), they did not observe reciprocal influences between autistic traits and affective problems over time: autistic traits at age 2 did not influence affective problems at age 3, nor did affective problems at age 2 influence autistic traits at age 3 after controlling for shared genetic and environmental influences.

To better characterize the role of autistic traits in human behavioral development, the present study was designed to prospectively examine relationships among early precursors of social behavioral outcome 1) beginning in infancy (18 months), 2) using specific developmental measures of quantitative autistic traits (QATs; described in *Methods*, below), and 3) utilizing a comprehensive analytic strategy to evaluate previously uncharacterized associations between QATs and traits of general psychopathology.^1^ Our primary aim was to evaluate shared genetic and environmental influences on QATs and other early trait-based manifestations of general psychopathology to clarify whether overlap is already present in infancy and, if so, to what degree. The developmental nature of the study design affords potential insights into psychiatric nosology by disentangling independent components of liability that might become confounded (by virtue of their interactions) over the course of development, thereby elucidating domains of overlap and non-overlap among psychopathological constructs (Krueger et al. 2016) and designating potential early intervention targets.

## Methods

### Participants

Three hundred and fourteen twins and their families participated in the Early Quantitative Characterization of Reciprocal Social Behavior study (ERSB), a longitudinal study characterizing the development of QATs from infancy through the toddler years. Autistic trait data related to this study have been published previously (cf. Marrus et al. 2015), but not as they pertain to developmental overlap between traits of autism and psychopathology. Of these 314 twins, 222 were retained for the duration of the study.^2^ The twins were epidemiologically ascertained from a record of all twin births that occurred in the state of Missouri between 2011 and 2013 (cf. Constantino et al. 2017). Recruitment protocol and participant characteristics are summarized in Online Resources 1 and 2.

All procedures performed in studies involving human participants were in accordance with the ethical standards of the institutional and/or national research committee and with the 1964 Helsinki declaration and its later amendments. Specifically, the WUSM Human Research Protection Office (HRPO; 201208010) and the State of Missouri Department of Health and Senior Services Institutional Review Board (State IRB Approval #1296) approved all study procedures. Informed consent was obtained from parents of twins who participated in the study.

### Measures

Measures were completed by the consenting individual. Descriptive statistics for all measures are reported in Table 1, and information regarding their established reliability and validity are reported in Online Resource 3.

**Table 1:** Descriptive statistics (mean, standard deviation, range, borderline clinical cut-offs and associated sample characteristics) for study measures

	Mean (SD)	Range	Borderline clinical cut-off (%ile)	Borderline clinical cut-off (score)	# ≥ cut-off	% ≥ cut-off
vrRSB						
SCI	20.1 (8.1)	4–65	84	28	45	14.3
RRB	1.7 (2.8)	0–19	84	5	30	9.6
RSB	21.8 (9.8)	6–82	84	31	43	13.7
BITSEA						
Problem	7.6 (5.0)	0–34	75	13 girls, 15 boys	38	12.1
Competence*	16.9 (2.9)	4–22	85	14	52	16.6
SRS						
SCI	24 (14.7)	0–115	84	58	4	1.8
RRB	3.2(3.9)	0–32	84	10	14	6.3
RSB	27.2 (17.9)	0–147	84	67	6	2.7
CBCL						
Internalizing	4.7 (4.6)	29–71	84	14	6	2.7
Externalizing	7.5 (6.8)	28–76	84	21	7	3.2

*Lower scores are of greater clinical concern

#### Video-Referenced Rating of Reciprocal Social Behavior

A video-referenced rating of Reciprocal Social Behavior (vrRSB; Marrus et al. 2015) was used to ascertain autistic traits at baseline. The vrRSB is described in detail by Marrus et al. (2015). Briefly, it is a 44-item quantitative autistic trait scale suitable for children 12 through 24 months of age. To help parents make nuanced evaluations of variations in early social communication, the vrRSB provides a 3-min video to serve as a ‘scoring anchor.’ In this video, a 19-month old child interacts with several adults. Throughout the video, critical early social behaviors (e.g., turn-taking, motivation to engage, responsiveness to social cues) are portrayed. The scoring anchor is designed to provide a naturalistic benchmark against which to evaluate early childhood behavior, thereby standardizing informants’ responses to items. Following the video, parents compare their child’s behavior to behavior portrayed in the video scoring anchor for 13 “video-referenced” items probing aspects of social communication and interaction. The remaining 31 “non-video-referenced” items also assess behaviors related to DSM5 clinically important domains of ASD, specifically, social communication and interaction (*SCI*) and restricted interests and repetitive behavior (*RRB*; Marrus et al. 2015). The *vrRSB Total* score quantifies reciprocal social behavior (RSB; hereafter, *vrRSB Total* will be referred to as *RSB*) and consists of *SCI* and *RRB* subscales. Higher *RSB, SCI,* and *RRB* scores indicate greater impairment. Importantly, *RSB* and *SCI* scores are continuously distributed, capturing trait variation in the general population (Marrus et al. 2015).

#### Brief Infant-Toddler Social and Emotional Assessment

The Brief Infant-Toddler Social and Emotional Assessment (BITSEA; Briggs-Gowan and Carter 2006) is a 42-item quantitative trait scale assessing social-emotional and behavioral milestones and delays in children one through three years of age. It is a well-established screening tool (Briggs-Gowan and Carter 2006; Briggs-Gowan et al. 2013), can be administered to parents as a questionnaire or as part of a structured interview, and takes roughly seven minutes to complete. The BITSEA generates two main scores: 1) a *Behavior Problem* score consisting of *Externalizing*, *Internalizing*, and *Dysregulation* subscales, and 2) a *Competence* score measuring key features of social adaptation, including social-emotional skills and aspects of social relatedness. Specifically, the BITSEA *Competence* score is comprised of 11 items assessing rule-abiding behavior, play behavior, attention, empathy, motivation to master new skills, and quality of peer interactions. Qualitatively, these items exhibit substantial overlap with the vrRSB *SCI* scale; however, *SCI* includes more items assessing communicative competency and social engagement.

#### Social Responsiveness Scale, Second Edition, Preschool Version

The Social Responsiveness Scale, second edition (SRS-2; Constantino and Gruber 2012) is a 65-item quantitative trait scale measuring quantitative autistic traits (QATs) from preschool through adulthood and requiring about 15–20 min to complete. The *SRS-2 Total* score quantifies QATs and encompasses both DSM5 criterion domains of ASD (*SCI* and *RRB*). Higher *Total* scores indicate greater impairment. In the present study, *SRS-2 Total* served as the primary outcome measure in prospective longitudinal predictions of QATs.

#### Child Behavior Checklist

The Achenbach Scales of Empirically Based Assessment (i.e., the Child Behavior Checklist, CBCL; Achenbach and Rescorla 2000) is an extensively validated parent- and/or teacher-report measure of behavior problems during preschool and childhood (Verhulst and Van der Ende 1992). The CBCL preschool forms (ages 1.5–5 years) yield seven syndrome scores (*Emotionally Reactive, Anxious/Depressed, Somatic Complaints, Withdrawn, Sleep Problems, Attention Problems,* and *Aggressive Behavior*) and five DSM-oriented scores (*Depressive, Anxiety, Autism Spectrum, Attention Deficit/Hyperactivity*, and *Oppositional Defiant Problems*). *Emotionally Reactive, Anxious/Depressed, Somatic Complaints,* and *Withdrawn* syndrome scores can be combined to create an *Internalizing* composite, and *Aggressive Behavior* and *Attention Problems* syndrome scores can be combined to create an *Externalizing* composite. In the present study, *Internalizing* and *Externalizing* composites served as the primary outcome measures in prospective longitudinal predictions of psychopathology. Although there is evidence to support their use as autism screeners, *Withdrawn* and *Autism Spectrum Problems* scores were not included in the present analyses due to low discriminative validity (area under the curve = 0.68; Havdahl et al. 2016) and lower sensitivity and specificity for autism relative to the SRS (Hampton and Strand 2015).

#### Goldsmith Child Zygosity Questionnaire

The Goldsmith Child Zygosity Questionnaire (Goldsmith 1991) is a 27-item parent-report measure developed to assess the degree of physical similarity between twins, from which determinations about zygosity can be made. Agreement with biological indicators of zygosity has been shown to exceed 93% (Price et al. 2000). In the present study, the Goldsmith Child Zygosity Questionnaire was administered over the phone to all families of same-sex twin pairs. Questionnaire-based zygosity determinations were genetically confirmed in 24 randomly-selected families; correspondence between questionnaire and genetic determinations was observed in all instances. Twins pairs were excluded from analyses (n_twin pairs_ = 6) if zygosity could not be determined by questionnaire.

### Data Analysis

All analyses were conducted in R (R Studio, 2016). First, Pearson product-moment correlations were computed to explore bivariate associations among BITSEA (*Behavior Problem, Competence, Internalizing, Externalizing, Dysregulation*) and vrRSB (*RSB, SCI, RRB*) subscales. Non-independence of twin observations was mitigated by randomly selecting one twin per family for inclusion in the primary set of analyses, with co-twins used for the purpose of quasi-replication. Next, we implemented exploratory factor analysis (EFA) as an initial step in empirically-deriving independent and overlapping domains of behavioral variation from assessment data collected at baseline. Subscales rather than individual items were entered into factor analyses to increase the ratio of number of observations to number of items. These subscales included *Externalizing, Internalizing, Dysregulation, Competence, SCI* (social communication and interaction)*,* and *RRB* (restricted interests and repetitive behavior) indices. Composite indices (i.e., *Behavior Problem* and *RSB*) were omitted to avoid item redundancy. As above, dependencies in twin data were accounted for by randomly selecting one twin per family to be included in analyses. Observations were evaluated via principal component and minimum residual factoring methods, followed by oblimin rotation. Tucker Lewis Index (TLI) values 0.90–0.92 were judged adequate fit, 0.92–0.95 good fit, and > 0.95 excellent fit (Marsh et al. 2004); root mean square error of approximation (RMSEA) values < 0.10 were judged adequate fit and < 0.08 good fit (Kline 1998). Differences between models were considered significant if a reduction in the Bayesian information criterion (BIC) greater than 5 points was observed (Kass and Raftery 1995).

Falconer’s formula (Falconer 1960) was used to provide estimates of broad heritability for early developmental domains (*SCI, Competence, Behavior Problem*): Heritability = 2(*r*_*MZ*_*– r*_*DZ*_). Within domains, heritability was estimated separately for male/male and female/female twin pairs, as well as for the entire sample. Although Falconer’s formula is commonly used to estimate heritability (Rice 2008), it overestimates genetic contributions to behavior in the context of nonadditive variance (Lykken et al. 1993). Thus, estimates of heritability were capped at monozygotic twin correlations in the present study.

Finally, hierarchical linear modeling (HLM) was used to 1) investigate domains of overlap during early development and 2) predict QATs, internalizing, and externalizing behaviors at 36 months. To account for twin dependency, family was modeled as a random effect.

## Results

### Correlation Analyses

To provide an indication of interrelationships among early developmental traits, Pearson product-moment correlations were calculated examining subscale-level associations at baseline between quantitative measures of general psychopathology (i.e., *Competence* and *Behavior Problem* indices, wherein the *Behavior Problem* index consisted of *Externalizing, Internalizing,* and *Dysregulation* subscales) and autistic traits (i.e., total *vrRSB* score*,* consisting of *SCI* and *RRB* subscales). As expected, *Competence* was not significantly correlated with *Behavior Problem* subscales, and both the total *vrRSB* score and *SCI* exhibited only weak correlations with *Behavior Problem* subscales (Table 2; see Online Resource 4 for co-twin analyses). Meanwhile, *Competence, SCI,* and total *vrRSB* score exhibited strong correlations with each other, and *Behavior Problem* subscales exhibited modest correlations with each other. *RRB* was significantly, albeit modestly, correlated with all scales. Importantly, this overall pattern of results suggests that *Competence, SCI,* and the total *vrRSB* score are highly similar to each other and highly distinct from other subscales.

**Table 2:** Pearson product-moment correlations (n_twins_ = 154) among measures of general psychopathology and QATs at 18 months

	Extl.	Intl.	Dysreg.	Behav. Probs	Comp.	SCI	RRB	RSB
Externalizing	1							
Internalizing	0.20*	1						
Dysregulation	0.36***	0.41***	1					
Behavior Problems	0.70***	0.65***	0.79***	1				
Competence	0.03	0.05	−0.04	0.05	1			
SCI	0.08	0.27***	0.18*	0.25**	0.70***	1		
RRB	0.24**	0.23**	0.37***	0.46***	0.26**	0.46***	1	
RSB	0.13	0.29***	0.26**	0.34***	0.66***	0.97***	0.67***	1

*** *p* < 0.001, ** *p* < 0.01, * *p* < 0.05; Ext. = externalizing, Intl. = internalizing, Dysreg. = dysregulation, Behav. Probs = Behavior Problems, Comp. = Competence

### Factor Structure at Baseline

To evaluate whether correlational associations reflected an underlying factor structure, vrRSB and BITSEA subscales were submitted to exploratory factor analyses (EFA). Results of EFA identified a two-factor solution wherein *SCI* and *Competence* comprised a “social adaptation” factor and *Externalizing, Internalizing, Dysregulation,* and *RRB* comprised a “problem behaviors” factor (Online Resources 5 & 6; see Online Resources 7 & 8 for co-twin analyses). Whereas *SCI* loaded strongly onto social adaptation (twin loading = 0.99, co-twins loading = 1.00) and weakly onto problem behaviors (twin loading = 0.05, co-twin loading = 0.02), factor loadings for *RRB* were equivocal (twin loadings = 0.36, 0.42 and co-twin loadings = 0.38, 0.37 for social adaptation and problem behaviors, respectively). In this sense, *RRB* diverged from *SCI* despite strong correlations between the two traits within individuals. In conjunction with the limited number of (*n* = 11) and variation in (SD = 2.8) *RRB* items in typically-developing children, this divergence informed our decision to use *SCI* as the primary index of autistic traits. The heritability of *RRB* is provided in Online Resource 9, and we note that its factor structure warrants exploration in future studies.

### Heritability

Having characterized factor structure, we aimed to determine whether the distinction between social adaptation and behavior problems may have emerged, in part, due to differences in patterns of genetic transmission. Thus, the heritabilities of *Competence, Behavior Problem,* and *SCI* indices were examined at baseline. Falconer’s estimates of heritability are reported in Table 3. Irrespective of gender, heritability estimates appeared roughly 2.5–3 times larger for *SCI* and *Competence* indices compared to the *Behavior Problem* index. The magnitude of these estimates warrants mention. Across all participants, the estimation of heritability for *SCI* was 0.90 and the estimation of heritability for *Competence* was 0.89, indicating that a large majority of variance in these traits can be attributed to genetic factors. In contrast, the estimation of heritability for the *Behavior Problem* index was 0.30, indicating that environmental factors account for a greater proportion of variance in this trait than genetic factors.

**Table 3:** Twin correlations (*r*_MZ_ and *r*_DZ_), associated confidence intervals (5%, 95%), and Falconer’s heritability estimates (H^2^) at 18 months

Subscale	Falconer’s heritability			
	n_pairs_	*r*_MZ_	*r*_DZ_	H^2^
SCI				
Male/Male	58	0.89 (0.77, 0.95)	0.30 (−0.06, 0.59)	0.89
Male/Female	36	NA	0.15 (−0.19, 0.45)	NA
Female/Female	56	0.91 (0.80, 0.96)	0.48 (0.15, 0.71)	0.85
All	150	0.90 (0.84, 0.94)	0.27 (0.08, 0.45)	0.90
Competence				
Male/Male	58	0.92 (0.83, 0.96)	0.29 (−0.07, 0.59)	0.92
Male/Female	36	NA	0.35 (0.03, 0.61)	NA
Female/Female	56	0.78 (0.56, 0.89)	0.38 (0.03, 0.65)	0.78
All	150	0.89 (0.81, 0.93)	0.37 (0.19, 0.53)	0.89
Behavior Problem				
Male/Male	58	0.82 (0.65, 0.91)	0.67 (0.41, 0.83)	0.31
Male/Female	36	NA	0.54 (0.26, 0.73)	NA
Female/Female	56	0.68 (0.41, 0.85)	0.55 (0.24, 0.76)	0.27
All	150	0.74 (0.59, 0.84)	0.59 (0.44, 0.71)	0.30

n_pairs_ = number of twin pairs; MZ = monozygotic; DZ = dizygotic; H^2^ = broad heritability

### Construct Overlap

Given the above results, suggesting a dissociation between *Behavior Problem* indices and *SCI* and *Competence*, we next investigated the extent to which quantitative risk factors for autism and general psychopathology are shared in infancy. To this end, we evaluated construct overlap among *SCI*, *Competence,* and *Behavior Problems* at baseline using hierarchical linear modeling (HLM; Online Resource 10). In the first regression, *Behavior Problem* and *Competence* indices were used to predict *SCI*. The *Behavior Problem* index was entered as the sole predictor in Model 1A, *Competence* was entered as the sole predictor in Model 1B, and both *Behavior Problem* and *Competence* indices were entered as predictors in Model 2. This incremental approach enabled calculation of the proportion of unique variance in *SCI* accounted for by *Behavior Problems* (marginal R^2^ Model 2 – unique R^2^ Model 1B) and *Competence* (marginal R^2^ Model 2 – unique R^2^ Model 1A). Results indicated that *Behavior Problems* accounted for 4% of unique variance in *SCI,* whereas *Competence* accounted for 48% of unique variance in *SCI*.

**Fig. 1:**
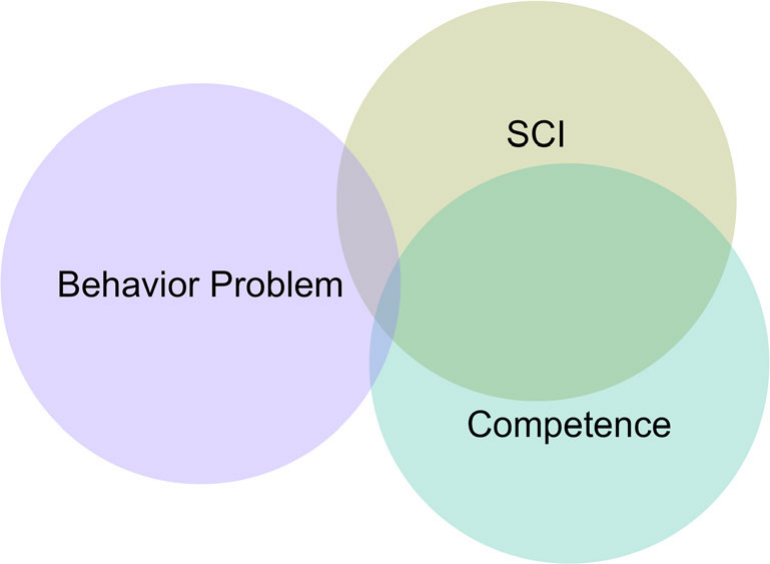
Venn diagram approximately to scale depicting construct overlap among *Behavior Problem, SCI,* and *Competence* indices. Overlap between *Behavior Problem* and *SCI* (4%) and *Behavior Problem* and *Competence* (1%) indices was minimal, whereas overlap between *Competence* and *SCI* indices was substantial (48%). These results emphasize the phenotypic similarity between *Competence* and *SCI*

We ran similar incremental analyses with *Competence* and *Behavior Problem* indices as our dependent variables (Online Resource 10)*.* Importantly, differences in unique R^2^ values were smaller when comparing incremental analyses within a given domain (e.g., Model 1B vs. Model 4A, both modeling overlap between *Competence* & *SCI*) relative to across domains (e.g., Model 1B vs. Model 2A, modeling overlap between *Competence* & *SCI* and *Problems* & *SCI*, respectively). This suggested that our estimates of overlap were reliable. To visualize subscale relationships, we created a Venn diagram depicting average estimates of overlap among *SCI, Competence,* and *Behavior Problem* indices (Fig. 1). In line with predictions, *SCI* and *Competence* exhibited considerable overlap, accounting for almost half of each other’s variance. Meanwhile, *SCI* and *Behavior Problem* indices and *Competence* and *Behavior Problem* indices exhibited considerably less overlap. These results provide converging evidence for the phenotypic similarity of traits assessed by *SCI* and *Competence*.

### Prospective Longitudinal Cross-Trait Prediction at 36 months

Despite relative non-overlap between *Behavior Problem* indices and *SCI* and *Competence* at 18 months, evidence indicates that such traits are inextricably linked in childhood (e.g., Hallett et al. 2010). To clarify the time course of autistic and psychopathologic quantitative trait overlap, we proceeded to investigate the temporal trait and cross-trait stabilities of *SCI, Competence, and Behavior Problem* indices. Specifically, we were interested whether infant measures of general psychopathology (i.e., the *Behavior Problem* index) would predict the severity of autistic traits during the toddler years (i.e., QATs; assessed by *SRS-2 Total*) above and beyond infant measures of social communicative autistic traits (i.e., *SCI*). Conversely, we were interested whether infant measures of social communicative autistic traits would predict the severity of internalizing and/or externalizing behavior during the toddler years (assessed by the CBCL; *Internalizing* and *Externalizing* subscales) above and beyond infant measures of general psychopathology. Finally, we were interested whether *Competence* would significantly improve predictive capacities above and beyond within-trait predictors. To this end, we ran incremental regressions using HLM. Within-trait predictors were entered into incremental models at Step 1, *Competence* was entered at Step 2, and cross-trait predictors were entered at Step 3. Independent variables were assessed at 18 months and dependent variables were assessed at 36 months.

Results are detailed in Online Resource 11. As expected, Step 1 (within-trait prediction) was uniformly significant, with *SCI* accounting for 26% of the variance in QATs and the *Behavior Problem* index accounting for 15% and 9% of the variances in *Internalizing* and *Externalizing* behavior, respectively. In Step 2, *Competence* accounted for a small albeit significant proportion of additional variance in QATs (unique R^2^ = 3%); *Competence* did not account for significant additional variance in either *Internalizing* or *Externalizing* behavior. Finally, in Step 3 (cross-trait prediction), the *Behavior Problem* index accounted for a small albeit significant proportion of additional variance in QATs (unique R^2^ = 2%); *SCI* did not account for significant additional variance in either *Internalizing* or *Externalizing* behavior. Given *Behavior Problem* and *RRB* indices clustered together in factor analyses, it remained unclear to what extent the *Behavior Problem* index improved prediction of QATs above and beyond comprehensive measures of autistic traits indexing both social communication and restricted, repetitive behaviors. To this end, a fourth incremental regression was run substituting *RSB,* a composite scale derived from *SCI* and *RRB*, for *SCI*. Under these conditions, *Behavior Problems* ceased to explain significant additional variance in QATs (χ^2^ = 3.11, *p* = 0.08).

**Fig. 2:**
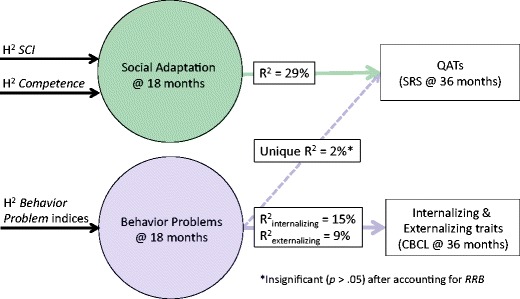
Prospective longitudinal prediction of QATs and general psychopathologic traits at 36 months from social adaptation (*Competence, SCI*) and behavior problems at 18 months. R^2^ = proportion of variance explained; unique R^2^ = proportion of incremental variance explained

Considering the above factor analyses, heritability analyses, and patterns of cross-trait overlap, these findings suggest that half of the causal influence on *Competence* is shared with that of *SCI* (Fig. 1), and that the construct jointly indexed by *SCI* and *Competence* accounts for about one third of the variation in autistic trait burden at 36 months (Fig. 2). Meanwhile, *Behavior Problem* indices exhibited separable mechanisms of causation relative to *Competence* and *SCI* and significantly predicted internalizing and externalizing traits at 36 months. Of note, correlations between autistic traits and internalizing (*r* = 0.52, *p* < 0.001) and externalizing (*r =* 0.30, *p* < 0.001) traits were roughly two times larger at 36 compared to 18 months, suggesting a strengthening relationship among these constructs over the course of early development.

## Discussion

To our knowledge, the present study is the first to assess standardized ratings of both early autistic traits and general psychopathologic traits 1) during the first two years of life and 2) in a prospective, genetically-informative sample, epidemiologically ascertained from the general population through birth records. Doing so allowed us to investigate the extent to which autistic and general psychopathologic traits—for which extreme variation constitutes clinical abnormality—overlap early in development. We observed a general pattern of independence of these constructs, which manifested highly disparate genetic and environmental structures. Furthermore, we observed that *Competence*, a well-known index of social adaptation measuring early developmental changes in social-emotional skills and social relatedness (e.g., empathy, play behavior, mastery motivation), exhibited substantial factorial and genetic overlap with variation in *SCI* (social communication and interaction)*,* which represents a core DSM5 criterion domain for autism. *SCI* accounted for almost half of variance in *Competence* at 18 months, and *SCI* and *Competence* demonstrated strong heritability. Importantly, we can be confident that rater bias was minimal given observations of high heritability, which are predicated on a single parent’s ability to provide discrepant data regarding non-identical siblings (see Zeeuw et al. 2017 for a discussion of rater bias in parent report measures), and the fact that study measures that were implemented have strong evidence for experimental validity against independent observational assessments (e.g., Briggs-Gowan and Carter 2008; Constantino and Gruber 2005; Keenan and Wakschlag 2000).

In contrast, early developmental precursors of internalizing and externalizing behaviors (*Behavior Problem* indices) were predominantly influenced by environmental rather than genetic factors in infancy and were only weakly correlated with *SCI*. The superordinate construct comprised of *SCI* and *Competence* explained roughly one-third of the variation in quantitative autistic traits at age 3 but did not improve explanatory power for internalizing or externalizing traits above and beyond *Behavior Problem* indices, reflecting its role as a major parameter of adaptation that is distinct and dissociable from general psychopathology. Interestingly, *RRB* (restricted interests and repetitive behavior) was more strongly correlated with *Behavior Problem* than *Competence* and *SCI* indices and segregated with general psychopathologic traits in exploratory factor analyses. Although such results are consistent with a theory of fractionation of autistic symptomatology (e.g., Happé et al. 2006), we defer interpretation of these findings about the role of *RRB* in development, given the instability of our factor analyses with respect to *RRB* (see Online Resources 7 & 8) and aforementioned psychometric limitations (e.g., limited number of and variation in *RRB* items) associated with the measurement of RRBs in the present normative sample. Further work with more comprehensive, RRB-specific measures—which may prove most informative in a sample enriched for children with elevations in autistic trait burden—is warranted to determine whether and how such behaviors influence the developmental course of trait overlap between risk factors for autism and general psychopathology.

Recasting the early psychological construct of *Competence* as one that arises from the same heritable influences that—at the extremes of trait aggregation within an individual—constitute social communicative deficits in autism represents a substantial shift in prevailing notions of psychological *Competence*. Specifically, it suggests that understanding the genetic origins of social communicative autistic trait variation is critical to understanding early behavioral adaptation in the general population. Extending recent advances from the field of autism genetics, which indicate that common genetic variants operating in an additive fashion incur heritable influences associated with autism spectrum disorder (Constantino et al. 2013; Gaugler et al. 2014), the present study supports the notion that such common variants represent heritable influences associated with social adaptation for all children. With respect to treatment implications, this raises the possibility that existing social communication interventions for autism may help resolve social and emotional impairments that often co-occur with psychopathology in the absence of categorical autism. However, further research in clinical populations is needed to evaluate the utility of such treatments.

Consistent with Micalizzi et al. (2016), results of the present study indicate that social communicative autistic impairment and general psychopathologic impairment are separable developmental domains with distinct genetic etiologies. Additionally, our results suggest that associations between autistic and psychopathologic traits strengthen over the course of early development (i.e., 18 to 36 months), bridging research in older samples that demonstrated strong correlations between degree of autistic impairment and degree of internalizing (Hallett et al. 2012) and externalizing (Ronald et al. 2010; Taylor et al. 2013) behaviors. Since studies in older children have identified shared genetic variance for autistic impairment and symptoms of general psychopathology (Hallett et al. 2012; Taylor et al. 2013; Ronald et al. 2008; Ronald et al. 2010), one way to reconcile the apparent discrepancy in causal overlap between studies of infants and studies of children is to infer that *non-specific* (general) psychopathologic symptoms can affect the severity of autistic syndromes (and vice versa) over the course of development—this would reflect the same phenomenon of phenotypic pleiotropy that was recently observed in a sibling study of autism recurrence (Mous et al. 2017) in which symptoms of ADHD and motor impairment exacerbated autistic severity in the context of inherited familial susceptibility to autism.

Importantly, whereas studies of trait overlap in older samples conflate shared genetic influences (i.e., genetic factor A directly influences both traits of autism and internalizing disorder traits) with longitudinal, interactive effects (i.e., a mediation model; genetic factor A influences traits of autism, which in turn influence internalizing disorder traits), studies in infant samples are well-positioned to disentangle these competing hypotheses. Integrating the present findings with extant literature, a pattern emerges wherein shared genetic variance is commonly observed during childhood but not infancy, suggesting a mediation model wherein genetic influences on autistic traits may exacerbate symptoms of general psychopathology over the course of development. Consistent with this notion, abnormalities in *Competence* during the toddler years predict psychiatric symptoms in elementary school (Briggs-Gowan and Carter 2008). If supported by subsequent research, this carries the implication that adverse interactions between autistic and psychopathologic liabilities might be preventable if recognized in infancy or early childhood. Alternatively, it is possible that genetic influences on autistic and psychopathologic traits take effect at different times.

Although the size of our twin sample was substantial and more than adequate to detect the associations described in this report, the derivation of more precise estimates of heritability using structural equation modeling was not possible at this sample size. Future studies of larger samples would be expected to replicate the heritability estimates obtained by Falconer’s formula. Sample size limitations also precluded item-level factor analyses on *Competence* and *SCI* subscales. Such analyses could provide more fine-grained information about the specific elements of *SCI* and *Competence* that contribute most strongly to their correlation, thereby designating effective targets for behavioral intervention. It bears mention, however, that there is heterogeneity in autistic syndromes. While this work supports measurable trait-level stability, heterogeneity implies the potential for different developmental mechanisms underlying autistic traits. For example, there are some autistic syndromes whose emergence is preceded by relatively normative development through the age of two years followed by severe regression. In such circumstances, the observed relationships may not apply. Finally, it remains unknown at what age interactive effects between social communicative autistic traits and traits of general psychopathology emerge. Future studies examining the relationship between these constructs over the course of early development, spanning infancy to early school age, are needed to fully characterize the time course and behavioral corollaries of trait overlap. This knowledge, in turn, may pinpoint critical periods of intervention during which negative interactions between autistic traits and traits of general psychopathology can be mitigated.

## Conclusions

The present study is the first to trace causal overlap between quantitative, social communicative autistic traits—assessed using a newly-validated measure of early reciprocal social behavior—and specific aspects of general psychopathologic traits to their developmental origins in infancy. At this early juncture, two established predictors of general psychopathology (social competence and problem behaviors) were independent and exhibited separable mechanisms of causation (the former highly heritable, the latter at this age strongly influenced by common environmental factors). Given that autistic trait variation in the general population is related to the genetic causes of autism itself (Ronald et al. 2006; Robinson et al. 2011; Constantino 2014), characterizing the early developmental time course of associations between autistic and psychiatric traits is essential to understanding a) the emergence of their well-documented trait overlap (in typically-developing populations), b) autism comorbidity (in clinical populations), and c) the factors that modulate enduring features of social behavioral adaptation in all children. We note that it would be consistent with these findings that the superimposition of independently-inherited psychopathologic trait liabilities upon a critical level of autistic trait liability might actually *contribute to the causation of autism itself (as a categorical clinical condition),* particularly in cases in which symptomatic features of joint (comorbid) liability remain appreciable over the course of later development. At a minimum, results of the present study indicate that the clinical comorbidity of specific psychiatric syndromes with autism may arise from interactions between autistic liability and independent susceptibilities to other psychopathologic traits. This suggests opportunities for preventive amelioration of outcomes of these interactions in infancy, perhaps via interventions to target developmentally-independent, behaviorally-modifiable traits (e.g., emotional dysregulation or attention problems—see Mous et al. 2017) that may conspire to exacerbate autistic severity over the course of development.

## Electronic supplementary material

ESM 1 (PDF 60.1 kb)

ESM 2 (PDF 70.3 kb)

ESM 3 (PDF 96.6 kb)

ESM 4 (PDF 56.9 kb)

ESM 5 (PDF 60.7 kb)

ESM 6 (PDF 71.2 kb)

ESM 7 (PDF 67.1 kb)

ESM 8 (PDF 74.6 kb)

ESM 9 (PDF 66.4 kb)

ESM 10 (PDF 84.2 kb)

ESM 11 (PDF 96.9 kb)

## Footnotes

1 Much like QATs, these general psychopathologic traits are influenced by the same underlying liability as their pathological counterparts (Spatola et al. 2010; Oord et al. 2003), providing an important vantage point from which to study early precursors of childhood and adolescent psychopathology. Although functional impairment from a singular pathologic trait typically only occurs at extreme levels of aggregation (e.g. 2 SD from the population mean), significant impairment can also arise from less extreme aggregation of two or more pathological traits within an individual.

2 Minor variations in sample size across analyses due to missing data are reported in Tables and Online Resources.
