# Supplementary material for: Early Origins of Autism Comorbidity: Neuropsychiatric Traits Correlated in Childhood Are Independent in Infancy
Source: J Abnorm Child Psychol. 2018 Mar 16;47(2):369–79. doi: 10.1007/s10802-018-0410-1 (PMC6139282; doi:10.1007/s10802-018-0410-1)
Supplement: Supplementary file 1 — (PDF 60.1 kb) [file 10802_2018_410_MOESM1_ESM.pdf]

Early origins of autism comorbidity: Neuropsychiatric traits correlated in childhood are independent in infancy, *Journal of Abnormal Child Psychology*

Online Resource 1

Participation was solicited via a letter describing the study, followed by a phone call. Phone numbers were requested in an initial mailing to each family. To be eligible to participate, we required consent from each twin's legal guardian/primary caregiver. The consenting individual was additionally required to speak fluent English (the only language spoken in upwards of 90% of Missouri homes; 'QuickFacts: Missouri,' 2011-2015[1]). A phone interview was conducted to collect demographic and medical information.

In sum, participation was discussed with 330 eligible families of twins in the specified age range during the calendar years 2011-2013, and every family who expressed interest was enrolled. Birth records were reviewed to determine whether there were differences between families that participated and those that did not. With the exception of maternal education, for which the study sample had a higher percentage of mothers with college and graduate degrees, no significant differences emerged.

[1] QuickFacts: Missouri. (2015). Available from: https://www.census.gov/quickfacts/table/PST045216/29 [12 May 2017]
