# Supplementary material for: Early Origins of Autism Comorbidity: Neuropsychiatric Traits Correlated in Childhood Are Independent in Infancy
Source: J Abnorm Child Psychol. 2018 Mar 16;47(2):369–79. doi: 10.1007/s10802-018-0410-1 (PMC6139282; doi:10.1007/s10802-018-0410-1)
Supplement: Supplementary file 2 — (PDF 70.3 kb) [file 10802_2018_410_MOESM2_ESM.pdf]

Early origins of autism comorbidity: Neuropsychiatric traits correlated in childhood are independent in infancy, *Journal of Abnormal Child Psychology*

Online Resource 2

Participant [percent of sample (%), number of individuals (n_{twins})] and response [mean, standard deviation (SD), range] characteristics at 18 and 36 months

	18 months		36 months	
	%	n_{twins}	%	n_{twins}
Gender				
Male	49.36	155	50.00	111
Female	50.64	159	50.00	111
Zygosity				
Monozygous	34.4	108	23.4	52
Dizygous	60.5	190	72.1	160
Same sex dizygous	37.6	118	44.1	98
Opposite sex dizygous	22.9	72	27.9	62
Unknown	5.1	16	4.5	10
Income				
<$29,999	16.6	52	8.1	18
$30,000-$59,999	25.5	80	14.4	32
$60,000-$89,999	24.2	76	18.0	40
$90,000-$119,999	17.8	56	15.3	34
$120,000-$149,999	7.6	24	4.5	10
$150,000-$179,999	2.5	8	4.5	10
$180,000-$209,999	2.5	8	4.5	10
$210,000-249,999	1.3	4	1.8	4
>$250,000	0.6	2	0.9	2
Unknown	1.3	4	27.9	62
Race				
American Indian/Alaska Native	0.0	0	0.0	0
Asian	1.3	4	1.8	4
Black/African-American	8.3	26	8.1	18
Caucasian	79.0	248	80.2	178
Native Hawaiian/Other Pacific Islander	0.0	0	0.0	0
Mixed race	10.8	34	9.9	22
Unknown/Other	0.6	2	0.0	0
Ethnicity				
Hispanic	7.0	22	5.4	12
Non-Hispanic	91.1	286	92.8	206
Unknown	1.9	6	1.8	4
	Mean (SD)	Range	Mean (SD)	Range
Age in months	18.68 (1.01)	17-23	35.69 (.61)	35-39
vrRSB (18 mo)/SRS-2 (36 mo)				
RSB (total) score	21.75 (9.81)	6-82	27.22 (17.9)	0-147
SCI	20.09 (8.1)	4-65	24.00 (14.7)	0-115

RRB	1.67 (2.81)	0-19	3.22 (3.86)	0-32
BITSEA (18 mo)/CBCL (36 mo)				
Behavior Prob/Externalizing	7.56 (5.01)	0-34	4.30 (4.47)	0-26
Competence/Internalizing	16.97 (2.95)	4-22	6.45 (6.16)	0-35

The gender ratio in this study did not differ compared to Missouri twins born between September and December 2011 and August and December of 2012, the source populations for the present sample (χ^2= 0.250, df = 1, p = .617). Median household income for the present sample was higher than the Missouri median of $47,202, falling between $60,000 and $89,999. Racial breakdown likewise differed from the Missouri population (χ^2=128.96, df = 3, p = .000; due to limited observations, American Indian/Alaska Native, Asian, Native Hawaiian/Other Pacific Islander, and Unknown/Other were merged into a single cell). Ethnic composition did not significantly differ (χ^2= 0.003, df = 1, p = .958). Raw scores are reported for behavioral assessments; RSB = reciprocal social behavior, SCI = social communication and interaction, RRB = restricted interests and repetitive behavior
