# Supplementary material for: Early Origins of Autism Comorbidity: Neuropsychiatric Traits Correlated in Childhood Are Independent in Infancy
Source: J Abnorm Child Psychol. 2018 Mar 16;47(2):369–79. doi: 10.1007/s10802-018-0410-1 (PMC6139282; doi:10.1007/s10802-018-0410-1)
Supplement: Supplementary file 3 — (PDF 96.6 kb) [file 10802_2018_410_MOESM3_ESM.pdf]

Early origins of autism comorbidity: Neuropsychiatric traits correlated in childhood are independent in infancy, *Journal of Abnormal Child Psychology*

Online Resource 3

Reliability and validity of the vrRSB: The vrRSB is brief, facilitating its inclusion in large-scale research studies, and exhibits strong psychometric properties (Marrus et al., 2015). Specifically, it has demonstrated 6-month test-retest reliability (ICC) of .70, clinical validity relative to both categorical screening measures of autism (e.g., Modified Checklist for Autism in Toddlers; M-CHAT) and to measures of language development (e.g., MacArthur-Bates Communicative Development Inventory), high heritability, and high internal consistency (α = .89 in the present sample). Although the M-CHAT was used to validate the vrRSB, floor effects in epidemiological samples made the M-CHAT less relevant to the present study, analyses for which depended on continuously distributed measurements of quantitative traits.

Reliablity and validity of the BITSEA: The BITSEA *Behavior Problem* and *Competence* scores exhibited good test-retest *(r* = .87 and .85, respectively) and inter-rater (α = .68 and .61, respectively) reliability and one-year stability (*r* = .65 and .53, respectively) in a large sample of healthy infants (Briggs-Gowan et al., 2004). Internal consistency (α) was .76 in the present sample. Reliability results were subsequently replicated in a sample of healthy infants (Briggs-Gowan & Carter, 2006). The clinical validity of the BITSEA *Behavior Problem* score has been established relative to the Preschool Age Psychiatric Assessment, a commonly-used diagnostic measure of early childhood psychopathology (correlations between related scales ranged from .57-.73; Briggs-Gowan et al., 2013), as well as a number of informant-report measures such as the Infant Toddler Social and Emotional Assessment, the Ages and Stages Questionnaires, and the CBCL (correlations between related scales ranged from .46-.77; Briggs-Gowan & Carter, 2006). Although the BITSEA *Competence* score was only modestly correlated with measures of psychopathology, it segregated with ASD diagnosis in a small case-control sample (n=33; Briggs-Gowan & Carter, 2006), predicted M-CHAT risk status (area under the curve ranged from .63-.71; Gardner et al., 2013), and identified infants at-risk for psychiatric disorders. For example, the BITSEA identified 67.9% of infants who went on to meet criteria for a psychiatric disorder in elementary school (Briggs-Gowan & Carter, 2008).

Reliability and validity of the SRS-2: Regarding reliability, the SRS-2 exhibits high internal consistency (i.e., > .90; Constantino et al., 2000; Bölte et al., 2008), test-retest reliability and temporal stability (i.e., > .80, with correlations slightly higher for clinical relative to non-clinical samples; Constantino et al., 2000; Bölte et al., 2008; Constantino et al., 2009), and interrater reliability (i.e., mother-father agreement > .90 in clinical samples and > .60 in normative samples; Constantino et al., 2003; Bölte et al., 2008). Comparable results for internal consistency (α = .94 and $\alpha = .95$) and parent/teacher interrater agreement (*r* = .77) were obtained in a preschool standardization sample aged 30-54 months (Constantino & Gruber, 2012). Internal consistency (α) was .95 in the present sample. Regarding screening utility, measures of sensitivity and specificity typically range from .78 to .90 and .67 to 1.0, respectively (Constantino & Gruber, 2005; Charman et al., 2007). Finally, concurrent validity has been established relative to commonly used diagnostic measures (e.g., Autism Diagnostic

Interview—Revised, Autism Diagnostic Observation Schedule; Constantino et al., 2003; Bölte et al., 2008; Charman et al., 2007) and behavioral assessments (e.g., Children's Communication Checklist, Social and Communication Disorders Checklist, and Childhood Autism Rating Scale; Charman et al., 2007; Pine, Guyer, Goldwin, Towbin, & Leibenluft, 2008).

Reliability and validity of the CBCL: Regarding reliability, the CBCL preschool forms (ages 1.5-5 years) exhibit high test-retest reliability (r = .85), interrater reliability (r = .61 between parents), and temporal stability (r = .61; Achenbach & Rescorla, 2000). Internal consistency in the present sample was also high (α = .93). Regarding validity, the preschool forms have been shown to differentiate between children referred for clinical assessment and controls with 84.2% accuracy (Achenbach & Rescorla, 2000), and evidence for construct validity has been obtained relative to the Infant-Toddler Social and Emotional Assessment ($.46 \leq r \leq .72$; Briggs-Gowan & Carter, 1998) and DSM criteria indexing symptoms of Oppositional Defiant and Conduct Disorders (r = .49; Keenan & Wakschlag, 2000).

References

Briggs-Gowan, M. J., & Carter, A. S. (1998). Preliminary acceptability and psychometrics of the infant–toddler social and emotional assessment (ITSEA): A new adult-report questionnaire. *Infant Mental Health Journal, 19*(4), 422–445.

Briggs-Gowan, M. J., Carter, A. S., Irwin, J. R., Wachtel, K., & Cicchetti, D. V. (2004). The brief infant-toddler social and emotional assessment: Screening for social-emotional problems and delays in competence. *Journal of Pediatric Psychology, 29*(2), 143–155.

Charman, T., Baird, G., Simonoff, E., Loucas, T., Chandler, S., Meldrum, D., & Pickles, A. (2007). Efficacy of three screening instruments in the identification of autistic-spectrum disorders. *The British Journal of Psychiatry, 191*(6), 554–559.

Constantino, J. N., Przybeck, T., Friesen, D., & Todd, R. D. (2000). Reciprocal social behavior in children with and without pervasive developmental disorders. *Journal of Developmental & Behavioral Pediatrics, 21*(1), 2–11.

Constantino, J. N., Davis, S. A., Todd, R. D., Schindler, M. K., Gross, M. M., Brophy, S. L., et al. (2003). Validation of a brief quantitative measure of autistic traits: comparison of the social responsiveness scale with the autism diagnostic interview-revised. *Journal of Autism and Developmental Disorders, 33*(4), 427–433.

Constantino, J. N., Abbacchi, A. M., Lavesser, P. D., Reed, H., Givens, L., Chiang, L., et al. (2009). Developmental course of autistic social impairment in males. *Development and Psychopathology, 21*(01), 127–138.

Gardner, L., Murphy, L., Campbell, J., Tylavsky, F., Palmer, F., & Graff, J. (2013). Screening accuracy for risk of autism spectrum disorder using the brief infant-toddler social and emotional assessment. *Research in Autism Spectrum Disorders, 7*(5), 591–600.

Pine, D. S., Guyer, A. E., Goldwin, M., Towbin, K. A., & Leibenluft, E. (2008). Autism spectrum disorder scale scores in pediatric mood and anxiety disorders. *Journal of the American Academy of Child & Adolescent Psychiatry, 47*(6), 652–661.
