# Supplementary material for: Early Origins of Autism Comorbidity: Neuropsychiatric Traits Correlated in Childhood Are Independent in Infancy
Source: J Abnorm Child Psychol. 2018 Mar 16;47(2):369–79. doi: 10.1007/s10802-018-0410-1 (PMC6139282; doi:10.1007/s10802-018-0410-1)
Supplement: Supplementary file 4 — (PDF 56.9 kb) [file 10802_2018_410_MOESM4_ESM.pdf]

Early origins of autism comorbidity: Neuropsychiatric traits correlated in childhood are independent in infancy, *Journal of Abnormal Child Psychology*

Online Resource 4

Pearson product-moment correlations (n_{twins} = 154) among measures of general psychopathology and QATs at baseline in co-twins

	Externalizing	Internalizing	Dysregulation	Behavior Problem	Competence	SCI	RRB	RSB
Externalizing	1							
Internalizing	0.13	1						
Dysregulation	0.54***	0.39***	1					
Behavior Problems	0.71***	0.59***	0.87***	1				
Competence	-0.02	0.13	0.08	0.14	1			
SCI	0.03	0.31***	0.24**	0.29***	0.74***	1		
RRB	0.25**	0.33***	0.38***	0.47***	0.31***	0.53***	1	
RSB	0.10	0.35***	0.31***	0.37***	0.70***	0.97***	0.72***	1

*** p < 0.001, ** p < 0.01, * p < 0.05
