# Supplementary material for: Early Origins of Autism Comorbidity: Neuropsychiatric Traits Correlated in Childhood Are Independent in Infancy
Source: J Abnorm Child Psychol. 2018 Mar 16;47(2):369–79. doi: 10.1007/s10802-018-0410-1 (PMC6139282; doi:10.1007/s10802-018-0410-1)
Supplement: Supplementary file 5 — (PDF 60.7 kb) [file 10802_2018_410_MOESM5_ESM.pdf]

Early origins of autism comorbidity: Neuropsychiatric traits correlated in childhood are independent in infancy, *Journal of Abnormal Child Psychology*

Online Resource 5

Results of exploratory factor analyses (n_{twins} = 154) on BITSEA and vrRSB subscales at 18 months

	χ2	Df	TLI	RMSEA	95% CI	RMSR	BIC	Cum. Var. (%)
1 factor	86.24	9	0.397	0.24	(.19, .28)	0.17	40.91	29
2 factor	4.42	4	0.993	0.03	[0.0, .13)	0.03	-15.73	50

χ2 = chi-square; Df = degrees of freedom; TLI = Tucker Lewis Index; RMSEA= root mean square error of approximation; 95% CI = 95% confidence interval; RMSR = root mean square of the residuals; BIC = Bayesian information criterion; Cum. Var. = cumulative variance
