# Supplementary material for: Early Origins of Autism Comorbidity: Neuropsychiatric Traits Correlated in Childhood Are Independent in Infancy
Source: J Abnorm Child Psychol. 2018 Mar 16;47(2):369–79. doi: 10.1007/s10802-018-0410-1 (PMC6139282; doi:10.1007/s10802-018-0410-1)

**Early origins of autism comorbidity: Neuropsychiatric traits correlated in childhood are independent in infancy, *Journal of Abnormal Child Psychology***

**Online Resource 6** Factor structure at baseline. The data were best described by a 2-factor model, with *SCI* and *Competence* comprising a "social adaptation" factor and *Dysregulation, Internalizing, Externalizing,* and *RRB* indices comprising a "behavior problems" factor

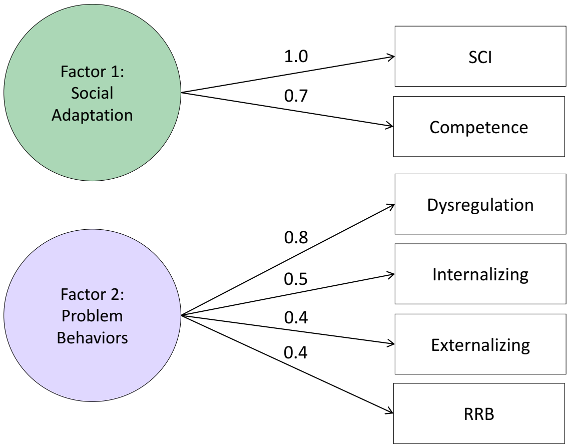

Supplement: Supplementary file 6 — (PDF 71.2 kb) [file 10802_2018_410_MOESM6_ESM.pdf]
