# Supplementary material for: Early Origins of Autism Comorbidity: Neuropsychiatric Traits Correlated in Childhood Are Independent in Infancy
Source: J Abnorm Child Psychol. 2018 Mar 16;47(2):369–79. doi: 10.1007/s10802-018-0410-1 (PMC6139282; doi:10.1007/s10802-018-0410-1)
Supplement: Supplementary file 7 — (PDF 67.1 kb) [file 10802_2018_410_MOESM7_ESM.pdf]

Early origins of autism comorbidity: Neuropsychiatric traits correlated in childhood are independent in infancy, *Journal of Abnormal Child Psychology*

Online Resource 7

Results of exploratory factor analyses (n_{twins} = 154) on BITSEA and vrRSB subscales at baseline in co-twins

	χ2	Df	TLI	RMSEA	95% CI	RMSR	BIC	Cumulative variance (%)
1 factor	112.31	9	0.390	0.276	(.23, .32)	0.18	66.86	33
2 factor	8.46	4	0.941	0.087	[0.0, .17)	0.04	-11.74	55

χ2 = chi-square; Df = degrees of freedom; TLI = Tucker Lewis Index; RMSEA= root mean square error of approximation; 95% CI = 95% confidence interval; RMSR = root mean square of the residuals; BIC = Bayesian information criterion
