# Supplementary material for: Early Origins of Autism Comorbidity: Neuropsychiatric Traits Correlated in Childhood Are Independent in Infancy
Source: J Abnorm Child Psychol. 2018 Mar 16;47(2):369–79. doi: 10.1007/s10802-018-0410-1 (PMC6139282; doi:10.1007/s10802-018-0410-1)

Early origins of autism comorbidity: Neuropsychiatric traits correlated in childhood are independent in infancy, *Journal of Abnormal Child Psychology*

Online Resource 8 Factor structure at baseline in co-twins. The data were best described by a 2-factor model, with *SCI, Competence,* and *RRB* comprising a "social adaptation" factor and *Dysregulation, Internalizing,* and *Externalizing* indices comprising a "behavior problems" factor. This structure is largely consistent with data from twins (Fig. 1), however in co-twins *RRB* loads onto "social competencies" rather than "behavior problems." Given psychometric limitations associated with measurement of *RRB* in very young typically-developing children (Marrus et al., 2015), instability was not altogether surprising, and *RRB* was excluded from subsequent analyses

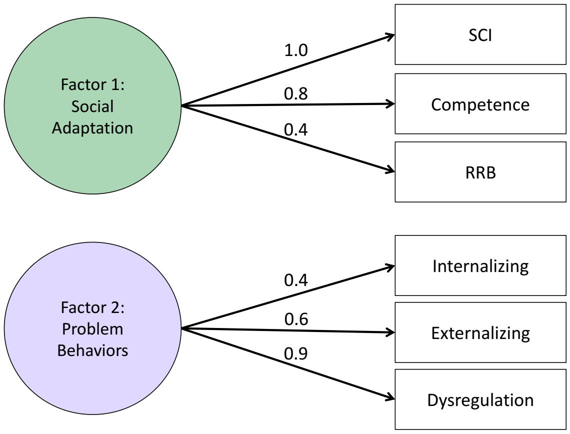

Supplement: Supplementary file 8 — (PDF 74.6 kb) [file 10802_2018_410_MOESM8_ESM.pdf]
