# Supplementary material for: Early Origins of Autism Comorbidity: Neuropsychiatric Traits Correlated in Childhood Are Independent in Infancy
Source: J Abnorm Child Psychol. 2018 Mar 16;47(2):369–79. doi: 10.1007/s10802-018-0410-1 (PMC6139282; doi:10.1007/s10802-018-0410-1)
Supplement: Supplementary file 9 — (PDF 66.4 kb) [file 10802_2018_410_MOESM9_ESM.pdf]

Early origins of autism comorbidity: Neuropsychiatric traits correlated in childhood are independent in infancy, *Journal of Abnormal Child Psychology*

Online Resource 9

Twin correlations (r_{MZ} and r_{DZ}), associated confidence intervals (5%, 95%), and Falconer's heritability estimates (H^2) for *RRB* at 18 months

	Falconer's heritability			
RRB	n_{pairs}	r_{MZ}	r_{DZ}	H^2
Male/Male	58	0.91 (.82, .96)	0.50 (.17, .72)	0.83
Male/Female	36	NA	0.09 (-.24, .41)	NA
Female/Female	56	0.48 (.13, .73)	0.15 (-.22, .48)	0.48
All	150	0.83 (.72, .89)	0.31 (.12, .48)	0.83

n_{pairs} = number of twin pairs; MZ = monozygotic; DZ = dizygotic; H^2 = broad heritability
