# Supplementary material for: Early Origins of Autism Comorbidity: Neuropsychiatric Traits Correlated in Childhood Are Independent in Infancy
Source: J Abnorm Child Psychol. 2018 Mar 16;47(2):369–79. doi: 10.1007/s10802-018-0410-1 (PMC6139282; doi:10.1007/s10802-018-0410-1)
Supplement: Supplementary file 10 — (PDF 84.2 kb) [file 10802_2018_410_MOESM10_ESM.pdf]

Early origins of autism comorbidity: Neuropsychiatric traits correlated in childhood are independent in infancy, *Journal of Abnormal Child Psychology*

Online Resource 10 Construct overlap among *SCI, Behavior Problems,* and *Competence* indices at 18 months

	SCI at 18 months			Behavior Problems at 18 months			Competence at 18 months		
	Model 1A: Problems	Model 1B: Competence	Model 2: Problems + Competence	Model 2A: SCI	Model 2B: Competence	Model 3: SCI + Competence	Model 4A: SCI	Model 4B: Problems	Model 5: SCI + Problems
Problems									
Estimate	0.29***	NA	0.21***	NA	NA	NA	NA	-0.11*	0.09*
SE	(.06)	NA	(.04)	NA	NA	NA	NA	(.06)	(.04)
Competence									
Estimate	NA	-0.72***	-0.70***	NA	-0.11*	0.16*	NA	NA	NA
SE	NA	(.04)	(.04)	NA	(.06)	(.08)	NA	NA	NA
SCI									
Estimate	NA	NA	NA	0.27***	NA	0.38***	-0.63***	NA	-0.71***
SE	NA	NA	NA	(.05)	NA	(.07)	(.04)	NA	(.04)
AIC	800.84	615.61	590.73	784.76	806.53	782.21	630.38	803.64	596.91
BIC	815.79	630.56	609.41	799.70	821.47	800.90	645.33	818.59	615.59
Log Likelihood	-396.42	-303.80	-290.36	-388.38	-399.26	-386.11	-311.19	-397.82	-293.45
Num. obs	310	310	310	310	310	310	310	310	310
Num. groups: Twin pairs	156	156	156	156	156	156	156	156	156
Var: Twin pairs (Intercept)	0.52	0.23	0.21	0.59	0.63	0.57	0.31	0.61	0.25
Var: Residual	0.40	0.25	0.23	0.34	0.36	0.34	0.23	0.37	0.21
Marginal R^2	0.08	0.52	0.56	0.07	0.01	0.08	0.49	0.01	0.50
Unique R^2	**0.04**	**0.48**	NA	**0.07**	**0.01**	NA	**0.49**	**0.01**	NA
Model comparisons	Mod 2 > Mod 1A, χ2 = 212.11*** Mod 2 > Mod 1B, χ2 = 26.881***			Mod 2 > Mod 1A, χ2 = 4.546* Mod 2 > Mod 1B, χ2 = 26.313***			Mod 2 > Mod 1A, χ2 = 5.0243* Mod 2 > Mod 1B, χ2 = 208.73***		

*** $p < 0.001$, ** $p < 0.01$, * $p < 0.05$; χ2 = chi-squared; marginal R^2 = variance accounted for by fixed factors (i.e., Problems, Competence); unique R^2 = non-overlapping variance. Unique R^2 values approximating overlap between SCI and Behavior Problems are coded blue, values approximating overlap between SCI and Competence are coded orange, and values approximating overlap between Behavior Problems and Competence are coded green
