# Supplementary material for: Early Origins of Autism Comorbidity: Neuropsychiatric Traits Correlated in Childhood Are Independent in Infancy
Source: J Abnorm Child Psychol. 2018 Mar 16;47(2):369–79. doi: 10.1007/s10802-018-0410-1 (PMC6139282; doi:10.1007/s10802-018-0410-1)
Supplement: Supplementary file 11 — (PDF 96.9 kb) [file 10802_2018_410_MOESM11_ESM.pdf]

Early origins of autism comorbidity: Neuropsychiatric traits correlated in childhood are independent in infancy, *Journal of Abnormal Child Psychology*

Online Resource 11 Longitudinal predictions of internalizing traits, externalizing traits, and QATs at 36 months from *Behavior Problem, Competence,* and *SCI* indices at 18 months

	1. Internalizing at 36 months			2. Externalizing at 36 months			3. QATs at 36 months			4. QATs at 36 months (post-hoc)		
	Step 1: Problems	Step 2: Problems + Competence	Step 3: Problems + SCI + Competence	Step 1: Problems	Step 2: Problems + Competence	Step 3: Problems + SCI + Competence	Step 1: SCI	Step 2: SCI + Competence	Step 3: Problems + SCI + Competence	Step 1: RSB	Step 2: RSB + Competence	Step 3: Problems + RSB + Competence
Problems												
Estimate	0.37***	0.37***	0.34***	0.30***	0.30***	0.30***	NA	NA	0.15**	NA	NA	0.11
SE	(.07)	(.07)	(.07)	(.07)	(.07)	(.07)	NA	NA	(.06)	NA	NA	(.06)
Competence												
Estimate	NA	-0.07	0.01	NA	0.00	-0.01	NA	-.22**	-.24**	NA	-.17*	-.19*
SE	NA	(.07)	(.09)	NA	(.07)	(.09)	NA	(.08)	(.08)	NA	(.08)	(.08)
SCI/ RSB												
Estimate	NA	NA	0.14	NA	NA	-0.02	0.55***	0.41***	0.35***	0.59***	0.49***	0.44***
SE	NA	NA	(.09)	NA	NA	(.09)	(.06)	(.08)	(.08)	(.06)	(.07)	(.08)
AIC	475.98	477.05	476.52	486.69	488.69	490.62	541.26	536.23	532.30	524.58	521.52	520.41
BIC	488.86	493.15	495.84	499.57	504.79	509.95	554.83	553.19	552.67	538.15	538.49	540.77
Log Likelihood	-233.99	-233.52	-232.26	-239.34	-239.34	-239.31	-266.6	-263.11	-260.15	-258.3	-255.76	-254.21
Num. obs	185	185	185	185	185	185	220	220	220	220	220	220
Num. groups: Twin pairs	96	96	96	96	96	96	111	111	111	111	111	111
Var: Twin pairs (Intercept)	0.33	0.35	0.34	0.40	0.40	0.40	0.37	0.37	0.30	0.30	0.31	0.27
Var: Residual	0.47	0.36	0.46	0.47	0.47	0.47	0.38	0.37	0.39	0.38	0.36	0.38
Marginal R^2	0.15	0.15	0.16	0.09	0.09	0.09	0.26	0.29	0.32	0.31	0.33	0.35
Model comparisons	Step 1 > Step 2 χ^2 = 0.9331	Step 2 > Step 3 χ^2 = 2.5267		Step 1 > Step 2 χ^2 = 0.0000	Step 2 > Step 3 χ^2 = 0.0626		Step 2 > Step 1 χ^2 = 7.0289**	Step 3 > Step 2 χ^2 = 5.9229*		Step 2 > Step 1 χ^2 = 5.0554*	Step 2 > Step 3 χ^2 = 3.1113	

*** $p < 0.001$, ** $p < 0.01$, * $p < 0.05$; χ^2 = chi-squared; marginal R^2 = variance accounted for by fixed factors (i.e., Problems, Competence, RSB)
